# Attention Deficit Hyperactivity Disorder (ADHD) Causes and Diagnosis in Adults: A Review

**DOI:** 10.7759/cureus.49144

**Published:** 2023-11-20

**Authors:** Bhagyesh Sapkale, Anupama Sawal

**Affiliations:** 1 Medicine, Jawaharlal Nehru Medical College, Datta Meghe Institute of Higher Education and Research, Wardha, IND; 2 Anatomy, Jawaharlal Nehru Medical College, Datta Meghe Institute of Higher Education and Research, Wardha, IND

**Keywords:** adhd treatment, complex attention deficit hyperactivity disorder (adhd), attention-deficit/hyperactivity disorder (adhd), adult adhd, attention deficit hyperactivity disorder (adhd)

## Abstract

This article focuses on attention deficit hyperactivity disorder (ADHD) in males of the adult age group, exploring its causes and diagnosis. ADHD is commonly identified in children and teenagers, often leading to academic difficulties. Diagnosing adult ADHD involves evaluating recent symptoms, assessing childhood history, examining functional impairment, obtaining developmental and mental health backgrounds, and ruling out other psychiatric conditions. The diagnostic assessment primarily relies on patient interviews, though input from family members and other sources can be valuable. Men and women show differences in ADHD symptoms and associated neurological conditions, with males more frequently diagnosed with ADHD. Inattention, hyperactivity, and impulsivity are signs of ADHD. The difficulties in identifying adult ADHD and separating it from behavioural problems are covered in the essay. It also explores the various symptoms of ADHD in children and adults and their impact on daily life. The causes of ADHD involve abnormalities in brain structure and function, as well as genetic factors. Treatment options for adult ADHD encompass medication, education, skill training, and psychological counselling. While medications can help manage symptoms, they do not provide a cure. The article concludes by emphasizing the need for a healthy lifestyle alongside therapy and medication to manage ADHD symptoms effectively.

## Introduction and background

Attention deficit hyperactivity disorder (ADHD) is frequently identified in kids or teenagers. Teachers frequently identify this issue before anyone else because having ADHD may contribute to poor academic achievement. So, a variety of ailments and conditions can have an impact on a student's academic performance. It's critical to correctly identify the source of the student's problems because ADHD is one of these potential causes. If you have ADHD, specific therapies can help [1]. Excessive impatience, mood swings, low self-esteem, sensitivity to rejection, stress, anxiety, trouble overcoming setbacks, procrastination, poor emotional control, and overwhelming feelings are some of the emotional symptoms associated with ADHD. Inattention, impulsivity, and hyperactivity are the hallmarks of ADHD, a chronic neurodevelopmental condition. Although it was once believed that this condition only affected children, it is now widely acknowledged that in a substantial number of cases, both symptoms and any resulting impairment will last into adulthood [2]. Similar to children with the disorder, adults with ADHD experience symptoms that are linked to significant interpersonal, professional, and educational problems. New drugs and psychotherapeutic techniques are being developed to achieve the best treatment outcomes in this group [3]. Procedures for diagnosing adults with ADHD use adult-standard rating measures to assess symptoms of ADHD that have occurred within the last six months.

Evaluate functional impairment in relationships, at work, school, and home. Obtain developmental history, including that from conception through childhood and education. Find out the family's mental health background, particularly concerning learning difficulties, attention and behaviour issues, ADHD, and tics. Ask about all of your first-degree relations. (Parents, siblings, and offspring). Conduct a physical check to rule out any underlying medical conditions (such as a severe head injury or seizures). Men and women are affected differently by the disease and the neurologic conditions frequently associated with ADHD. Men and boys are more likely to be observed than women and girls to receive an ADHD diagnosis. "Differences in symptoms may account for why males are diagnosed with ADHD more commonly. Males are likelier to exhibit impulsive or restless behaviour, while females are likelier to exhibit inattention and emotional instability too" [4]. The diagnostic evaluation in adulthood mainly relies on interacting with the patient. Direct communication with the patient is an essential part of the diagnostic process. Still, a thorough evaluation frequently combines techniques to reduce reporting bias and improve diagnosis accuracy, particularly when there may be outside incentives, like getting medication. To determine whether ADHD symptoms are causing functional impairment, physicians collect data in several domains. This entails assessing one's educational background, professional performance, interpersonal interactions, and self-care routines. Furthermore, evaluating time management, emotional stability, driving abilities, and general lifestyle offers a thorough grasp of how ADHD affects day-to-day functioning. A complete and accurate assessment of functional impairment in various circumstances is ensured using collateral information from various sources, including friends and family. Structured or semi-structured interviews, clinical judgment checklists, and questionnaires for specific disorders to learn how patients perceive themselves and how parents and teachers see them are all beneficial diagnostic techniques. Without a doubt, it's critical to take into account mental comorbidities when diagnosing ADHD, as well as to be aware of potential reference illnesses like bipolar disorder and borderline personality disorder (BPD) [5]. These disorders may have symptoms that are similar to ADHD, making diagnosis difficult. It's critical to distinguish between bipolar disorder and ADHD because, while both conditions may involve mood dysregulation, they require different kinds of care. Similar to this, it's critical to comprehend the characteristics of BPD because BPD's impulsivity and emotional instability might be confused with signs of ADHD.

The inability to focus on a task or activity is a defining trait of the inattentive type of ADHD. They need a better track record of keeping their promises. They frequently abandon unfinished tasks because they quickly grow bored, jumping from one partly-started project to another. Not only that, but they might need help to concentrate on just one item at a time [6]. Neuropsychological tests, which evaluate memory, attention, and executive function, offer unbiased perceptions of cognitive capacities and help validate cognitive impairments linked to ADHD. With the use of an organized methodology that incorporates self-report and supplementary data, diagnostic interviews such as DIVA methodically examine symptoms of ADHD and their implications. DIVA and similar tools follow defined standards and allow for a complete diagnosis based on large amounts of data. Clinicians obtain a comprehensive assessment that incorporates both subjective and objective measures by incorporating these assessments into the diagnostic procedure. This method improves knowledge about the nature of ADHD symptoms and how they affect a person's overall performance. Symptoms of ADHD may include inattention, hyperactivity, and impulsivity as follows: The symptoms of ADHD in children and adults might vary. Children not paying enough attention can have trouble focusing, make careless mistakes, get quickly bored or distracted, forget things, and find it difficult to finish assignments. Symptoms of hyperactivity can include restlessness, talking too much, trouble sitting seated, and excessive running or climbing. Children who exhibit impulsivity may exhibit impatience, frequent interruptions, and actions taken without considering their repercussions. Adults who suffer from inattention may find it difficult to focus, make thoughtless mistakes, struggle with organization and time management, avoid tasks requiring extended mental effort, misplace or lose items frequently, and change jobs or careers frequently. Adults with hyperactivity may exhibit restless feelings, an incessant need for stimulation, excessive talking, and trouble with sitting still tasks. Impulsivity symptoms can include impulsively making decisions without considering consequences, difficulty controlling strong emotional reactions, engaging in risky behaviours, and frequently changing plans or goals [7]. There is no specific test for ADHD. Typically, screening involves a physical examination to pinpoint the ailment or conditions generating symptoms, followed by a discussion. You will be asked questions about your actions and activity level [8]. Executive dysfunction/inattention was included in the Diagnostic and Statistical Manual of Mental Disorders, Fifth Edition (DSM-5) inattentive-only, hyperactive/impulsive-only, and combined presentations, but hyperactivity-only was limited to hyperactivity without high symptoms of impulsivity [9].

## Review

Search methodology

**Figure 1:**
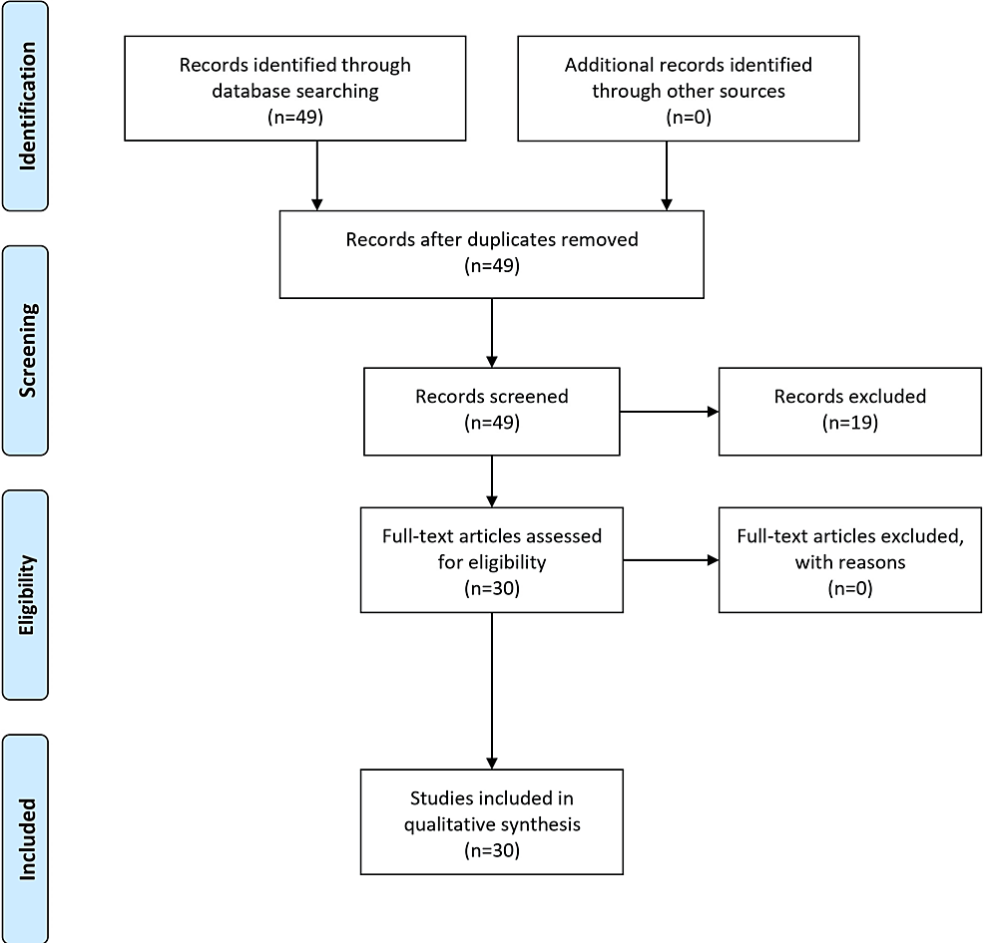
PRISMA flow diagram PRISMA: Preferred Reporting Items for Systematic Reviews and Meta-Analyses

To curate the information for this article about ADHD, we conducted a meticulous literature review. Our approach was systematic to gain a comprehensive understanding of the condition. Our research involved searching the National Institute of Mental Health's online archives and databases such as PubMed, PsycINFO, and Google Scholar. We utilized various search terms, such as "ADHD," "Attention Deficit Hyperactivity Disorder," "ADHD symptoms," "ADHD in adults," "ADHD in children," "ADHD diagnosis," and "ADHD treatment." To extract relevant research and publications and to focus our search, we used phrases like "AND" and "OR." We focused our research on articles from the past 10 years to ensure the accuracy of our data. However, we also considered critical early studies that laid the foundation for later studies or were extensively mentioned in recent works. We incorporated reviews, meta-analyses, and case studies using quantitative and qualitative data for a thorough understanding. Based on how relevant they were to the signs, causes, diagnosis, and treatments of ADHD, we chose the studies and publications we included in this review. We concentrated on the research findings in this area because the essay concentrated on gender differences in the diagnosis and manifestation of the disease. To maintain the credibility of our sources, we prioritized peer-reviewed articles. We conducted extensive research on ADHD by consulting with respected authorities and organizations such as the American Psychiatric Association, the World Health Organization, and the National Institute of Mental Health. Our research wasn't limited to academic articles but also included policies, fact sheets, and position papers. The figures presented in the article were likely derived from data from some sources we reviewed. To ensure a comprehensive overview, we carefully integrated the information from the figures into the text. Our search process was designed to guarantee the accuracy and completeness of our article in depicting ADHD in the current scientific landscape. The Preferred Reporting Items for Systematic Reviews and Meta-Analyses (PRISMA) flow diagram is shown in Figure 1.

An excessive degree of inattention, hyperactivity, and impulsivity that is pervasive, damaging in several circumstances, and frequently age-inappropriate are the hallmarks of ADHD, a neurodevelopmental condition. Problems like poor academic performance in childhood might be caused by not paying attention. ADHD is associated with some neurodevelopmental and mental illnesses and a few non-psychiatric conditions, which can cause further harm, especially in contemporary culture. The ability to sustain an excessively long and high degree of attention for things that are exciting or rewarding is known as hyperfocus. While people with ADHD find it challenging to focus on projects they are not particularly interested in accomplishing, they can often do so. Millions of children suffer from the chronic ailment known as ADHD, which usually lasts into adulthood. Chronic concerns such as difficulty focusing, hyperactivity, and impulsive conduct are symptoms of ADHD [10]. When it causes disruption or problems with schooling, ADHD is usually identified in youngsters in the school age range. Due to differences in how the symptoms manifest, it is frequently diagnosed in men rather than women. Girls are likelier to display passivity, whereas boys are likelier to display hyperactivity and other externalizing characteristics [11]. Many children are assigned female at birth, and adults with the condition also go undiagnosed [12]. ADHD is a neurodevelopmental disorder. It's not certain that it's a chronic condition, as certain strands of literature and statistical data show that in some people, ADHD or its symptoms and deficits don't carry over into adulthood.

Symptoms of ADHD

**Figure 2:**
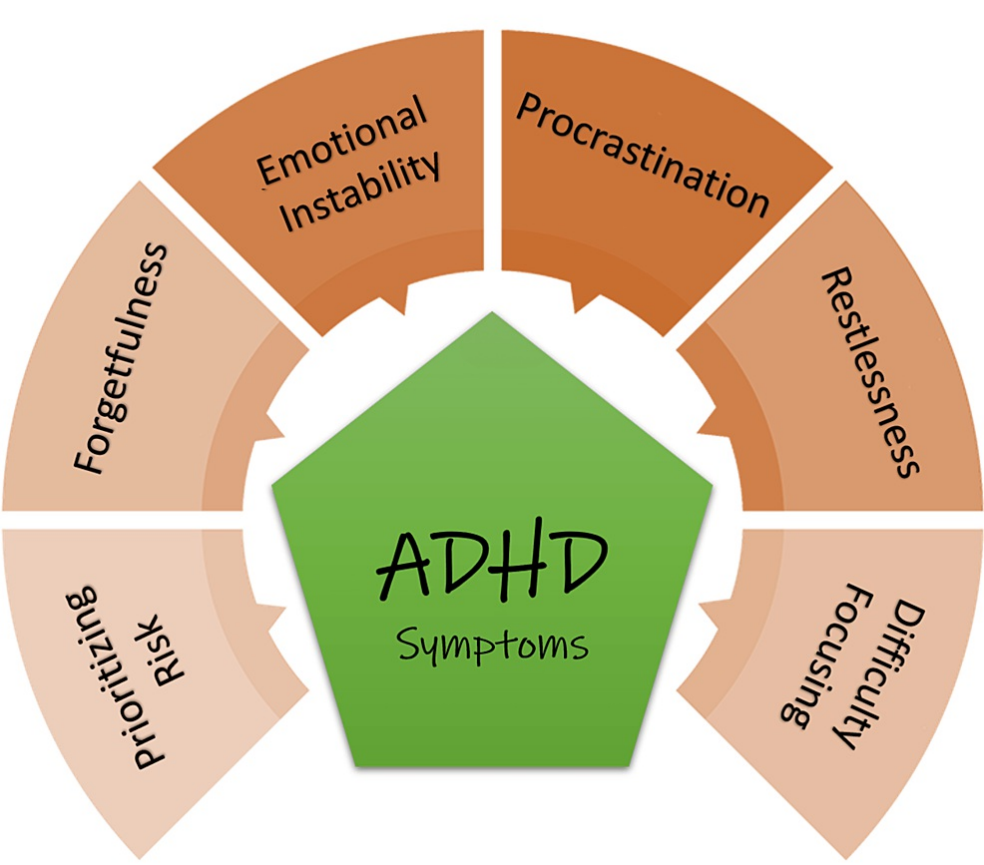
Common Symptoms of Attention Deficit Hyperactivity Disorder (ADHD) Reference: [13-18] Figure created by the authors.

Two categories of behavioural disorders can be used to categorize the symptoms of ADHD: inattention (trouble concentrating and focusing) and hyperactivity. Although not always the case, ADHD individuals experience problems that fall under both headings. Mood swings, low self-esteem, sensitivity to rejection, stress, anxiety, trouble overcoming setbacks, procrastination, poor emotional control, and overwhelming feelings are some of the emotional symptoms associated with ADHD [13]. Males are more likely than females to be diagnosed with ADHD [14]. Common symptoms of ADHD are shown in Figure 2.

Symptoms in Children and Teenagers

It is widely known that ADHD can be seen in children and teenagers before they turn six years old. It may be noticeable in different environments, like school and home. These symptoms can include inattention, hyperactivity, impulsivity, or a combination of these behaviours [15]. People with ADHD are more likely to abuse substances, especially in adolescence and adulthood. This is because people with ADHD are naturally impulsive and risk-takers. Studies show that the impulsive behaviour of people with ADHD increases the likelihood of drug or alcohol experimentation. In addition to abusing drugs, people with ADHD might also engage in risky behaviours such as driving recklessly, having risky sexual relationships, and doing physically demanding activities [16-17]. Inattention means having difficulty focusing, a short attention span, and a tendency to get distracted easily. Inattention can lead to mistakes, such as errors in homework, carelessness, or losing things. Additionally, completing tedious tasks or following instructions can take much work. As a result, schoolwork can be challenging when an assignment or task is constantly changing.

Hyperactivity and Impulsiveness

Difficult to sit quietly, especially in quiet or peaceful environments; prone to fidgeting; excessively active physically; very talkative; unable to wait their time; impulsive behaviour; and interfering with conversations are some of the main symptoms of impulsivity and hyperactivity, which make it difficult to focus at work. The feeling of risk is minimal or nonexistent. Adolescents and teenagers with ADHD may still have symptoms as adults [16]. As the demands of adulthood increase, adults, for instance, tend to decrease their hyperactivity while maintaining their inattentiveness [17]. Additionally, adult ADHD symptoms are far less severe than those in children. Absence of focus and carelessness [18], poor organizational abilities, difficulty focusing or prioritizing, frequent missing or misplacing of items, forgetfulness, agitation, and jitteriness, difficulty speaking in silence and speaking out of turn, blurting out responses, and frequently interrupting others mood swings, irritability, a quick temper, an inability to handle stress, extreme impatience, and risk-taking in activities, frequently with little or no thought beforehand are all signs of someone who lacks patience [14]. Mood swings and easy irritability are derived from emotional instability.

Causes of ADHD

Scientists have identified particular brain activity and structural abnormalities in individuals diagnosed with ADHD. The frontal lobe, located behind the forehead, organizes thoughts, decision-making, language usage, and behaviour control through "directed attention." Compared to those without ADHD, people with the disorder tend to display slower brain growth, which affects their ability to delay automatic attention. This skill is particularly challenging for individuals with ADHD [19]. Scientists have identified brain activity and structural abnormalities in individuals who have ADHD. Indeed, individuals with ADHD can pay attention; it's just that they have trouble focusing that attention on appropriate stimuli at the correct moment [20]. This difficulty is related to their inability to efficiently filter stimuli, making it difficult to stay motivated when confronted with a particular stimulus. It emphasizes how complex attention difficulties in ADHD involve problems with filtering, choosing stimuli, and maintaining motivation. While researchers have uncovered these brain changes, they are still trying to understand why they occur and contribute to ADHD symptoms [21]. However, recent evidence suggests that genetics may play a crucial role, as ADHD often runs in families. The heritability of ADHD can range from 70 to 80% [22]. Other potential causes and risk factors for ADHD include lead poisoning, brain anatomy, substance abuse during pregnancy, premature delivery, and low birth weight.

Diagnosis of ADHD

Adult ADHD diagnosis is more challenging due to disputes wondering whether the same set of symptoms used to assess toddlers and teenagers may also be utilized to diagnose adults. Adults with ADHD are most frequently diagnosed with depression, anxiety disorders, bipolar disorder, substance use disorders (SUDs), and personality disorders [23]. The diagnostic standards for children with ADHD indicate that at least five signs of inattention or five or more of the characteristics of hyperactivity and impulsivity in adults must be present to make the diagnosis [24]. Adults with ADHD must exhibit symptoms that moderate affect various elements of their lives, including [25]. School performance decreases, and driving is not the proper failure to keep family relations and friendship relationship issues. ADHD diagnosis aspects are shown below in Table 1.

**Table 1:** Attention Deficit Hyperactivity Disorder (ADHD) Diagnosis Aspects The co-diagnosis of depression, anxiety disorders, bipolar disorder, substance use disorders (SUDs), and personality disorders is a prevalent problem for adults with ADHD [23]. Adults who meet the essential criteria for identifying children with ADHD have at least five symptoms of inattentiveness, hyperactivity, and impulsivity [24]. Furthermore, the diagnosis must consider how these symptoms affect the person's life, including a noticeable reduction in performance at work or school, issues with driving, difficulties maintaining relationships with family and friends, and broader relational issues [25].

Topic/Aspect	Details/Explanation
Co-diagnosis with ADHD in Adults	Depression and anxiety are frequently diagnosed in adults with ADHD, bipolar disorder, SUDs, personality disorders, and ADHD in Adults diseases.
Symptom Criteria for Adult ADHD Diagnosis	An adult may be given an ADHD diagnosis if they show five or more of the inattentiveness symptoms listed under Adult ADHD, or five or more of the hyperactivity and impulsivity symptoms listed under Adult ADHD, as described for children with ADHD.
Impact of ADHD Symptoms in Adults	For an ADHD diagnosis in adults, symptoms should moderately impact multiple life aspects, such as: 1) Declined school performance 2) Inefficient driving 3) Inability to maintain relationships with family and friends 4) Relationship issues.

Treatment of ADHD

Treatments for adult ADHD frequently combine medication, instruction, skill development, and psychological counselling. Usually, a mix of these is the best course of action [23]. Although these drugs can help with many ADHD symptoms, they cannot treat the condition.

Medications

The most common therapies for ADHD are stimulants like methylphenidate or medicines containing amphetamine, while other drugs may also be used. Stimulants elevate and maintain neurotransmitter levels in the brain [26]. Antidepressants like bupropion and the non-stimulant atomoxetine are other therapies for ADHD [26,27]. Bupropion is superior to placebo and effective for the treatment of ADHD in adults [27]. A variety of techniques are used in psychological counselling for ADHD, such as behaviour therapy, parent education, social skills training, mindfulness-based therapies, cognitive-behavioural therapy (CBT), and supportive counselling. Atomoxetine and antidepressants act more slowly than stimulants. Still, they may be good alternatives if you cannot take stimulants due to health issues or if stimulants create significant adverse effects [28].

Psychological Counselling

**Figure 3:**
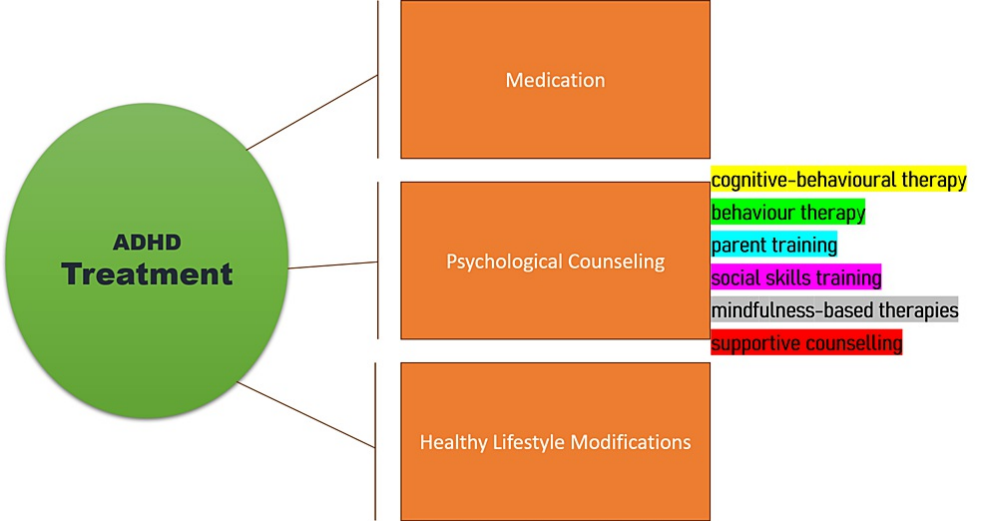
Attention Deficit Hyperactivity Disorder (ADHD) Treatment References: [23-30] Figure created by the authors.

Psychological counselling (also known as psychotherapy), education on the condition, and acquiring success-enhancing skills are frequently included in adult ADHD counselling. If neglected during childhood, ADHD may increase the likelihood of developing mental illness as an adult. ADHD treatments are shown in Figure 3.

Psychotherapy may be beneficial: managing time improves; temper should be managed appropriately; impulsive behaviour should be controlled; dealing with failures in social life; Increasing self-confidence, strengthening family relationships, and ability to answer questions and problems by solving them [29]. All children must maintain good health, but those who have ADHD must do so much more. Your kid can manage ADHD symptoms with behavioural treatment, medication, and a healthy lifestyle. Here are some good habits that might be helpful [30]. Creating healthy eating habits, such as consuming enough amounts of one, can reduce their daily exposure to screens from televisions, laptops, phones, and other devices by eating more fruits, vegetables, whole grains, and lean protein sources and engaging in age-appropriate daily physical activity and obtaining the recommended amount of sleep for your age each night. A summary table of the studies included is mentioned in Table 2.

**Table 2:** A summary table of the studies included ADHD: Attention-Deficit/Hyperactivity Disorder, NHS-UK: National Health Service UK, BMC: BioMed Central, PLoS One: Public Library of Science One

Author	Year	Summarized Result Details
Karceski et al. [1].	2016	A publication addressing the identification of ADHD. Continuing on the topic, the article discusses essential findings in the field, shedding light on critical aspects of ADHD diagnosis.
Johnson et al. [2].	2020	Insights into ADHD in adults are presented as crucial information for non-specialists in the British Journal of Hospital Medicine. The publication in Volume 81 offers valuable details to provide a comprehensive understanding of ADHD in adult populations.
Weiss et al. [3].	2003	Evaluation and management strategies for attention-deficit hyperactivity disorder in adults are explored in the Canadian Medical Association Journal. Continuing the discussion, the article in Volume 168 delves into practical approaches for handling ADHD in the adult population.
Brainandlife (Website) [4].	2020	Neurologists gain valuable insights from treating adults with ADHD, as accessed on April 6, 2023. Further exploration reveals the practical knowledge and experiences neurologists acquire while providing care to adults with ADHD.
Banaschewski et al. [5].	2017	Exploration of ADHD is presented in the Deutsche Arzteblatt International. Building on this exploration, the details contained in the publication (Volume 114) contribute to a deeper understanding of ADHD within the medical community.
Diamond et al. [6].	2005	Investigation into attention-deficit disorder, a neurobiologically and behaviourally distinct disorder, is conducted in Developmental Psychopathology. Continuing this line of inquiry, the publication in Volume 17 elucidates the differences between attention-deficit disorder and ADHD, providing valuable insights.
Goodman et al. [7].	2013	Comprehensive information on Attention-Deficit/Hyperactivity Disorder is available in the American Journal of Medicine. Elaborating on this comprehensive information found in Volume 309, the article addresses various aspects of ADHD, contributing to a holistic understanding of the disorder.
Medlineplus (Website) [8].	2023	Information on ADHD screening is accessible as of August 8, 2023. Continuing this discussion, the accessibility of information on screening methods facilitates early detection and treatment for those who may be at risk of ADHD.
Adler et al. [9].	2017	The structural aspects of adult ADHD are discussed in the International Journal of Methods in Psychiatric Research. Building on this discussion, the insights presented in Volume 26 aid in enhancing comprehension of the structural aspects of ADHD in adults.
Mayoclinic (Website) [10].	2019	Details on symptoms and causes of attention-deficit/hyperactivity disorder in children are accessed on July 8, 2023. Expanding on this information, the details contribute to a comprehensive understanding of the early manifestations and potential causes of ADHD in children.
Psychiatry (Website) [11].	2022	Insights into ADHD were accessed on July 8, 2023. Delving deeper into these insights, the article provides valuable information that enhances the overall awareness and understanding of ADHD.
Healthline (Website) [12].	2021	Fourteen symptoms of adult ADHD, from forgetfulness to fatigue, were accessed on July 8, 2023. Continuing on this topic, the identification and recognition of these symptoms contribute to a nuanced understanding of adult ADHD.
Salvi et al. [13].	2019	Exploration of clinical subtypes and associated characteristics of ADHD in adults is presented in Rivista di Psichiatria. Building on this exploration, the details in Volume 54 provide valuable insights into the diverse clinical presentations and characteristics of adult ADHD.
NHS-UK (Website) [14].	2017	Information on ADHD symptoms was accessed on July 8, 2023. Expanding on this information, the article delves into the various symptoms associated with ADHD, aiding in recognizing and diagnosing the disorder.
Ivanov et al. [15].	2018	A fifteen-year longitudinal investigation on early adult outcomes in children with ADHD and childhood serotonergic function is published in the European Neuropsychopharmacology. Continuing this longitudinal study, the findings in Volume 28 throw light on the long-term effects of serotonergic function in childhood on the outcomes of ADHD patients.
Kok et al. [16].	2020	A systematic literature review on the female side of pharmacotherapy for ADHD is available in PLoS One. Expanding on this review, the details in Volume 15 provide insights into the specific considerations and outcomes of pharmacotherapy for females with ADHD.
Oerbeck et al. [17].	2019	The correlation between adult ADHD symptoms and satisfaction with life, considering age and sex, is explored in the Journal of Attention Disorders. Continuing this exploration, the article in Volume 23 delves into the multifaceted relationship between ADHD symptoms, age, sex, and overall life satisfaction.
Oerbeck et al. [18].	2015	Psychiatry Research looks at the connection between adult ADHD symptoms, compulsive buying, impulse buying, depression, and past illegal drug use. Building on this examination, the details in Volume 228 provide a nuanced understanding of the interconnected factors influencing adult ADHD symptoms.
Nylander et al. [19].	2020	Five-year outcomes of ADHD diagnosed in adulthood are detailed in the Scandinavian Journal of Psychology. Continuing this longitudinal study, the findings in Volume 62 provide insights into the long-term outcomes and challenges faced by individuals diagnosed with ADHD in adulthood.
Cleveland Clinic (Website) [20].	2023	Information on Attention-Deficit/Hyperactivity Disorder was accessed on July 8, 2023. Expanding on this information, the article provides an overview of ADHD, enhancing awareness and understanding of the disorder.
Klusek et al. [21].	2022	The American Journal of Intellectual and Developmental Disabilities publishes an examination of ADHD in male adolescents and young adults with Fragile X syndrome. Continuing this examination, the details in Volume 127 contribute to a nuanced understanding of the interplay between ADHD and Fragile X syndrome in this specific population.
Rasmussen et al. [22].	2022	The overlooked aspect of ADHD in adults is discussed in Ugeskrift for Laeger. Continuing this discussion, the article in Volume 184 sheds light on the challenges and implications of overlooking ADHD in adults within the medical field.
Katzman et al. [23].	2017	The clinical implications of comorbid disorders in adult ADHD are discussed in BMC Psychiatry. Building on this discussion, the details in Volume 17 provide insights into the challenges and considerations associated with comorbidities in adults with ADHD.
NHS-UK (Website) [24].	2018	Insights into the attention deficit hyperactivity disorder diagnosis were accessed on July 9, 2023. Continuing this exploration, the article provides valuable insights into the diagnostic process for ADHD, contributing to improved identification and understanding.
Halleröd et al. [25].	2015	Consequences experienced by individuals diagnosed with ADHD as adults are explored in BMC Psychiatry. Expanding on this exploration, the details in Volume 15 shed light on the psychosocial and personal consequences experienced by adults following an ADHD diagnosis.
Mayoclinic(Website) [26].	2023	Information on the diagnosis and treatment of ADHD was accessed on July 8, 2023. Continuing this information, the article contributes to understanding the diagnostic and therapeutic approaches for managing ADHD in adults.
Sleath et al. [27].	2014	Communication about ADHD and its treatment during pediatric asthma visits is explored in the Community Mental Health Journal. Building on this exploration, the details in Volume 50 provide insights into the challenges and opportunities for communication regarding ADHD during pediatric asthma visits.
Kooij et al. [28].	2010	BMC Psychiatry contains a detailed consensus statement on the diagnosis and treatment of adult ADHD from the European Network Adult ADHD. Continuing this consensus statement, the details in Volume 10 contribute to standardized approaches for diagnosing and treating adult ADHD.
Knouse et al. [29].	2008	Expert Review of Neurotherapeutics discusses recent advances in the psychosocial treatment of adult ADHD. Expanding on this discussion, the details in Volume 8 provide insights into emerging methods of psychosocial therapy for individuals with ADHD.
Healthline (Website) [30].	2019	Information on ADHD treatment options was accessed on July 8, 2023. Continuing this information, the article provides an overview of available treatment options, facilitating informed decision-making for individuals with ADHD.

## Conclusions

The complexity of ADHD in adults is highlighted in this review, with a particular emphasis on men. ADHD is frequently diagnosed in childhood and can continue throughout maturity, posing special issues. An extensive evaluation is necessary to diagnose adult ADHD, taking into account functional impairment, childhood history, recent symptoms, and mental health histories. The presentation of symptoms varies by gender, with males being diagnosed more often. The paper highlights the value of collateral data, family involvement, and patient interviews in diagnosis. In addition to genetic variables, defects in the structure and function of the brain are the causes of ADHD. Although the exact cause is still being investigated, new data points to a genetic component. ADHD significantly negatively influences relationships, employment, education, and general well-being in daily life.

The illness is made more complex by emotional symptoms like mood swings, low self-esteem, and rejection sensitivity. A thorough evaluation is required for the diagnosis procedure, which takes into account not only the symptoms of ADHD but also the exclusion of other mental illnesses. It's critical to distinguish ADHD from comorbid conditions, including bipolar illness and borderline personality disorder, as well as behavioural issues. Various diagnostic instruments, such as checklists, neuropsychological testing, and interviews, aid in a thorough evaluation. The difficulties in diagnosing adult ADHD are also discussed in this review, with a focus on the need to overcome reporting biases and take outside motivations for medication seeking into account. Adults with inattention, hyperactivity, and impulsivity have distinct symptoms, necessitating a complicated diagnostic process. Since there is no particular test for ADHD, a comprehensive assessment is required, taking into account the patient's family history, developmental history, and other comorbidities.

The multimodal treatment approach for adult ADHD, which includes medication, education, skill training, and psychological counselling, is highlighted in the article's conclusion. Stimulant drugs do not provide a cure, but they can assist in controlling symptoms. Effective symptom management requires treatment, medicine, and a healthy lifestyle. Significantly, untreated ADHD increases the chance of developing comorbid mental health conditions later on. Adults with ADHD frequently have co-diagnoses with depression, anxiety disorders, bipolar illness, substance use disorders, and personality disorders. Acknowledging and treating ADHD in adulthood is essential for managing symptoms as well as averting future mental health issues. A comprehensive and individualized approach to diagnosis and treatment is critical for people navigating the challenges associated with adult ADHD.
